# Desmin Reorganization by Stimuli Inducing Oxidative Stress and Electrophiles: Role of Its Single Cysteine Residue

**DOI:** 10.3390/antiox12091703

**Published:** 2023-08-31

**Authors:** Diego Moneo-Corcuera, Álvaro Viedma-Poyatos, Konstantinos Stamatakis, Dolores Pérez-Sala

**Affiliations:** 1 Department of Structural and Chemical Biology, Centro de Investigaciones Biológicas Margarita Salas (CSIC), 28040 Madrid, Spain; moneodiego@gmail.com (D.M.-C.); aviedmapoyatos@gmail.com (Á.V.-P.); 2 Departamento de Biología Molecular, Universidad Autónoma de Madrid (UAM), 28049 Madrid, Spain; kstamatakis@cbm.csic.es; 3 Centro de Biología Molecular Severo Ochoa (UAM/CSIC), 28049 Madrid, Spain

**Keywords:** cysteine modification, intermediate filaments, cytoskeletal organization, oxidative stress, protein lipoxidation

## Abstract

The type III intermediate filament proteins vimentin and GFAP are modulated by oxidants and electrophiles, mainly through perturbation of their single cysteine residues. Desmin, the type III intermediate filament protein specific to muscle cells, is critical for muscle homeostasis, playing a key role in sarcomere organization and mitochondrial function. Here, we have studied the impact of oxidants and cysteine-reactive agents on desmin behavior. Our results show that several reactive species and drugs induce covalent modifications of desmin in vitro, of which its single cysteine residue, C333, is an important target. Moreover, stimuli eliciting oxidative stress or lipoxidation, including H_2_O_2_, 15-deoxy-prostaglandin J_2_, and CoCl_2_-elicited chemical hypoxia, provoke desmin disorganization in H9c2 rat cardiomyoblasts transfected with wild-type desmin, which is partially attenuated in cells expressing a C333S mutant. Notably, in cells lacking other cytoplasmic intermediate filaments, network formation by desmin C333S appears less efficient than that of desmin wt, especially when these proteins are expressed as fluorescent fusion constructs. Nevertheless, in these cells, the desmin C333S organization is also protected from disruption by oxidants. Taken together, our results indicate that desmin is a target for oxidative and electrophilic stress, which elicit desmin remodeling conditioned by the presence of its single cysteine residue.

## 1. Introduction

Intermediate filaments are cytoskeletal elements that influence cell architecture, organization, signaling, and gene regulation [1]. The type III intermediate filament protein family is integrated by vimentin, desmin, glial fibrillary acidic protein (GFAP), and peripherin. These proteins share considerable homology among them and between species, and their expression is tissue-selective, with vimentin being expressed mainly in cells of mesenchymal origin, desmin in muscle cells, GFAP in astrocytes, and peripherin in neurons of the peripheral nervous system [2]. From a structural point of view, type III intermediate filament protein monomers are constituted by a central, mainly helical, rod domain flanked by intrinsically disordered head and tail domains. During their complex assembly, parallel dimers form antiparallel tetramers, a number of which associate laterally and then head to tail to form filaments [3]. Type III intermediate filaments form extended networks in the cytoplasm of cells, where they play important functions not only in cell structure and mechanics but also in organelle homeostasis, cell migration, cytoskeletal interplay, and cell metabolism [4,5]. Moreover, type III intermediate filaments are involved in the cellular response to various types of stress [6,7,8,9,10].

**Figure 1:**
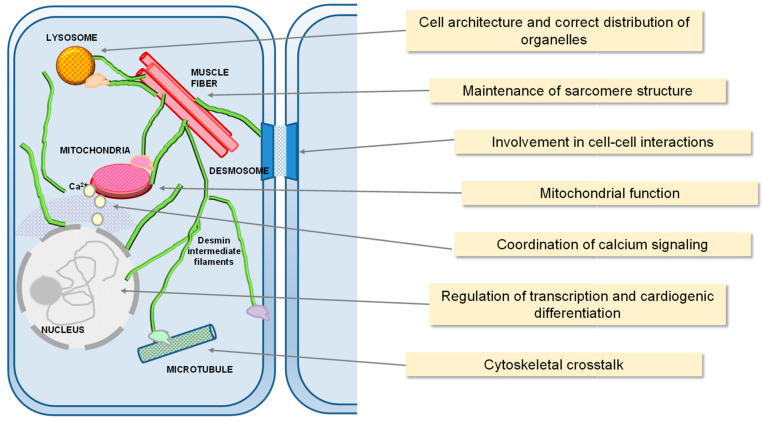
Schematic view of some of the cellular functions of desmin. Desmin plays a critical role in cell organization. It interacts, either directly or through cytolinkers or adaptor proteins, with multiple cellular structures and organelles, regulating their position and homeostasis. Among other functions, it maintains the structure of sarcomeres and their coupling to mitochondria; it can play roles in gene expression by participating in transcription factor complexes; and it participates in cell-cell contacts important for tissue integrity. For details, please see the text and references therein.

Desmin, a 53.5 kD protein composed of 470 amino acids encoded by the DES gene, is the main constituent of the muscle intermediate filament cytoplasmic cytoskeleton [11], and plays key roles in cell architecture and mechanics and in mitochondrial function, in cardiac, skeletal and smooth muscle [12]. Desmin is more abundant in the cardiomyocytes of mammals (2% of total protein) than in skeletal muscle (0.35%) or smooth muscle cells [13]. It is one of the first muscle-specific proteins to be expressed during myogenesis, as early as embryonic day 7.5 in the precardiac area and on day 9 in the myotomes and the smooth muscle cells of the mouse embryo (reviewed in [12,14]). Such early expression of desmin indicates its importance in cardiomyocyte development and function. Indeed, although desmin is not essential since desmin deficient or knockout mice are viable, these mice develop myopathies, especially cardiomyopathies, early in their lifetime [15,16]. Desmin-deficient mice develop altered muscle architecture, disorganized muscle fibers, skeletal and smooth muscle lesions, cardiac hypertrophy, and heart failure [17,18,19]. Moreover, cardiomyocytes from these mice demonstrate abnormal mitochondrial morphology, protein composition, distribution, and function, as well as increased oxidative stress [20,21,22]. Furthermore, desmin mutations have been associated with severe muscle pathologies, known as desminopathies, which can affect both skeletal and cardiac muscle [23], causing muscle weakness and/or cardiomyopathies and heart failure due to abnormal cytoskeletal organization or deposits of misfolded proteins [13,19,24]. Therefore, desmin plays an important role not only in cell mechanical resistance and force transmission in muscle but also in the correct distribution and function of organelles such as mitochondria and the nucleus [11]. Indeed, desmin has been recently shown to participate in the coordination of calcium signaling in skeletal muscle and to interact directly with mitochondria directly [25,26]. The complex and varied functions of desmin have been the subject of several excellent reviews [1,2,11,27] and are schematized in Figure 1.

Numerous pathologies related to desmin dysfunction are associated with oxidative stress, and a bidirectional relationship can exist between these phenomena. Indeed, muscle proteins from patients with desminopathies show features of oxidation and aggregation [28], and desmin itself has been shown to be oxidized and nitrated in affected muscles in myotilinopathies and desminopathies [29]. Moreover, oxidized desmin is more susceptible to cleavage [30], which could contribute to muscle waste, and is potentially also more prone to aggregate, since N-acetyl cysteine prevents stress-induced desmin aggregation in several models of desminopathies [28,31,32]. The interplay of desmin with mitochondria may have important implications for oxidative stress. Desmin dysfunction or mutation can elicit mitochondrial alterations. For instance, in heart failure, desmin filament breakdown precedes mitochondrial dysfunction and oxidative stress [33], which have important pathophysiological consequences (reviewed in [27,34]). Reciprocally, several pathophysiological situations including hypoxia, inflammation, and mitochondrial diseases can lead to oxidative stress [34,35,36], eliciting desmin modifications and potentially misfolding. Interestingly, desmin has been identified as one of the proteins nitrated in tyrosine in muscle biopsies from patients with mitochondrial diseases (mitochondrial respiratory chain dysfunction of various origins) [37].

Oxidative stress frequently augments peroxidation of unsaturated lipids, giving rise to an increase in electrophilic lipid species that can covalently adduct proteins, a process known as lipoxidation, which exerts important functional consequences [38]. Although low levels of electrophilic lipids can occur physiologically and be balanced by cellular antioxidant defenses, high levels can cause cellular and tissue damage and be involved in pathophysiology. For instance, the aldehyde acrolein can induce myocardial dysfunction in mice and form adducts with several proteins including desmin [39]. In addition, numerous drugs or their metabolites, including antineoplastic drugs such as doxorubicin, as well as certain non-steroidal anti-inflammatory agents, antibiotics, and antivirals, are able to induce oxidative stress and/or modify proteins directly and exert deleterious effects on muscle cells, often appearing as cardiotoxicity, in which desmin can be altered [40].

Interestingly, type III intermediate filaments possess a conserved cysteine residue, which is the only cysteine residue in vimentin, desmin, and GFAP, located in the rod 2B domain, which, in GFAP and vimentin, has been shown to be the target for a wide array of oxidative and electrophilic modifications, resulting in functional regulation of filament assembly or severe disruption of the cellular intermediate filament network (reviewed in [8]. Interestingly, mutants of vimentin and GFAP in which the single cysteine residue has been substituted by serine or alanine show increased resistance to disruption by numerous electrophilic agents [41,42,43], for which this residue has been proposed as a sensor for oxidative and electrophilic stress [10,41]. Importantly, given the high homology among type III intermediate filament proteins, a similar role could be hypothesized for the single cysteine residue of desmin, C333. Interestingly, several modifications of desmin C333 have been reported, including its involvement in the formation of oligomers in vitro in response to oxidative crosslinking [44]. In addition, disulfide-bonded desmin oligomers have been observed in a model of isolated rat hearts perfused with H_2_O_2_-containing buffer [45], as well as in isolated rat cardiomyocytes treated with H_2_O_2_ or diamide [46,47]. Moreover, desmin has been identified as a protein candidate for sulfenylation in isolated rat hearts treated with H_2_O_2_ [48]. Furthermore, an increase in desmin glutathionylation has been reported in HL-1 cardiomyocytes treated with H_2_O_2_ [49], and in a model of oxygen/glucose deprivation with subsequent replenishment in the presence of palmitate [50]. Nevertheless, the role of the modifications of this residue in the reorganization of the desmin network in response to various agents has not been established.

In this work, we sought to explore the effect of oxidative conditions on desmin modification and organization in various experimental models. Our results indicate that the desmin single cysteine residue, C333, is the target for several oxidative and electrophilic modifications in vitro and in cells. Moreover, cellular desmin organization undergoes a marked disruption upon cell treatment with oxidants and electrophiles, as well as in a model of chemical hypoxia, which is associated with oxidative stress. These alterations are attenuated in a desmin C333S mutant, suggesting the involvement of this residue in oxidative stress-elicited desmin cytoskeleton rearrangement.

## 2. Materials and Methods

### 2.1. Materials

Briefly, 15-deoxy-Δ^12,14^-prostaglandin J_2_ (15d-PGJ_2_) and its biotinylated analog (15d-PGJ_2_-B) were from Cayman Chemical (Ann Arbor, MI, USA). Oligonucleotides were from IDT (Coralville, IA, USA). Mini and maxiprep kits were from Roche (Basel, Switzerland) and NZyTech (Lisbon, Portugal), respectively. Bacteria used for transformations were from Invitrogen and NZyTech. Horseradish peroxidase (HRP)-coupled with streptavidin was from Cytiva (Amersham, UK). Doxorubicin, ethacrynic acid, chlorambucil, phenylarsine oxide, and As_2_O_3_ were from Merck (Tres Cantos, Spain). *N*-acetyl-*p*-benzoquinone imine (NAPQI) and biotinylated iodoacetamide ((+)-Biotin-(PEO)3-iodoacetamide) were from Santa Cruz Biotechnology (Santa Cruz, CA, USA). BioGEE (glutathione ethyl ester biotin amide) was from Invitrogen (Alcobendas, Spain). Anti-desmin antibodies, clone DE-U-10 (D-1033), and rabbit polyclonal ab15200 were from Merck and Abcam (Cambridge, UK), respectively. HRP-conjugated secondary antibodies were from Dako (Glostrup, Denmark).

### 2.2. Methods

In vitro modification of desmin. Purified recombinant human desmin wt (accession number AAA99221.1) and the C333S mutant were obtained from Abvance Biotech SL (Madrid, Spain). The recombinant proteins in 8 M urea, 5 mM Tris-HCl pH 7.6, 1 mM EDTA, 10 mM 2-mercaptoethanol, 0.4 mM PMSF, and approximately 0.2 M KCl were subjected to a process of ultrafiltration and sequential dialysis, as previously reported for vimentin [51,52], in order to achieve refolding and removal of EDTA. Protein concentration was estimated from its A280 nm using an extinction coefficient of 22,450 M^−1^·cm^−1^, and the protein was kept in aliquots at −80 °C until use. For the assessment of the effect of various compounds on desmin in vitro, incubation mixtures containing 4 µM desmin in 5 mM Pipes-Na, pH 7.0, 0.1 mM DTT, and the indicated concentrations of the various agents were prepared. Assays were carried out at room temperature or at 37 °C, as indicated. At the end of the incubations, aliquots were subjected to SDS-PAGE under reducing or non-reducing conditions. The incorporation of biotinylated reagents was assessed by protein transfer to PVDF membranes (Immobilon-P), incubation of blots with HRP-streptavidin, and ECL detection. The protein was visualized by western blot with anti-desmin antibody (monoclonal DE-U-10 at 1:5000 dilution) or by total protein staining on membranes with Gel Code (ThermoFisher Scientific, Alcobendas, Spain) or on gels with Sypro Ruby (Bio-Rad, Hercules, CA, USA) and transillumination under UV light, as previously described [53].

Cell culture and treatments. H9c2 cardiomyoblasts (rat heart myoblasts derived from embryonic BD1X rat heart myocardium) from the European Collection of Authenticated Cell Cultures (ECACC General Cell Collection, originally deposited by the ATCC) were cultured in DMEM plus antibiotics (100 U/mL penicillin, 100 μg/mL streptomycin), supplemented with FBS (10% *v*/*v*, Gibco, New York, NY, USA), routinely in a humidified atmosphere with 5% CO_2_. Cells were seeded on 6-well cell culture plates containing one coverslip at the bottom of each well. For assessment of the effects of oxidants and electrophiles, H9c2 cardiomyoblasts at approximately 80% confluence were transiently transfected with pCMV-desmin wt or C333S, and 48 h later, incubated in serum-free medium in the presence of vehicle (H_2_O or DMSO at 0.1% (*v*/*v*), as required), 1 mM H_2_O_2_ for 1 h, 10 µM 15d-PGJ_2_ for 2 h or CoCl_2_ at 50 or 100 µM for 24 h. Treatment with the various drugs was also carried out in serum-free medium at the concentrations indicated in the figure legends. At the end of the experiment, coverslips were removed and fixed for immunofluorescence, and cells in the plate were processed for protein analysis. For induction of chemical hypoxia, cells cultured on coverslips transfected as above were treated in serum-free medium in the presence of 50 or 100 µM CoCl_2_ for 24 h and fixed. SW13/cl.2 adrenal carcinoma cells devoid of cytoplasmic intermediate filaments were the generous gift of Prof. A. Sarriá (University of Zaragoza, Zaragoza, Spain) and have been previously described [54,55].

Plasmids and transfections. The plasmid pCMV6-AC-Desmin coding for human desmin (NM_001927) was from Origene Technologies (Rockville, MD, USA). The plasmid mEmerald-Desmin-C-18, coding for human desmin (accession number NM_001927) fused to mEmerald, was a gift from Michael Davidson (Addgene plasmid # 54059; http://n2t.net/addgene:54059, accessed on 14 December 2020.; RRID: Addgene 54059). Mutants of both constructs coding for desmin C333S were obtained by site-directed mutagenesis with the kit from NZyTech, following the instructions from the manufacturer and using oligos: 5′-CAGATCCAGTCCTACACCTCCGAGATTGACGCCCTCAAGGGCAC-3′ and the complementary reverse. The plasmids coding for human desmin were used in all experiments, given that human and rat desmin protein sequences are 98% identical. H9c2 or SW13/cl.2 cells were transiently transfected using mixtures of Lipofectamine 2000 and DNA in Optimem (1 µg of DNA plus 3 µL of Lipofectamine 2000 per well of a p6 plate, or 1.8 µg of DNA plus 4.5 µL of Lipofectamine 2000 per p60 dish). Transfection and recovery were performed in a serum-containing medium in the absence of antibiotics. Unless otherwise indicated, cells were analyzed and/or subjected to treatments 48 h after transfection.

Cell lysis and western blot. Routinely, cells in cultured dishes were washed with ice-cold PBS before being lysed by scraping in 50 mM Tris-HCl pH 7.5, 0.1 mM EDTA, 0.1 mM EGTA, 0.5% (*w*/*v*) SDS, 50 mM sodium fluoride, 0.1 mM sodium orthovanadate, and protease inhibitors: 2 μg/mL leupeptin, 2 μg/mL aprotinin, 2 μg/mL trypsin inhibitor, and 1.3 mM Pefablock, and passing cell suspensions through a narrow needle (26 1/2G). Protein concentration in lysates was measured with the bicinchoninic acid (BCA) protein quantification kit (ThermoFisher Scientific). Lysates were denatured in Laemmli buffer (80 mM Tris-HCl pH 6.8, 2% (*w*/*v*) SDS, 10% (*v*/*v*) glycerol, 0.15% (*w*/*v*) bromophenol blue), with or without 5% (*v*/*v*) β-mercaptoethanol, for 5 min at 95 °C, and aliquots containing 20 μg of protein were loaded onto 12.5% (*w*/*v*) SDS-polyacrylamide gels. Proteins were transferred to Immobilon-P membranes (Millipore, Burlington, MA, USA) using a semidry system (Bio-Rad), and membranes were processed for the detection of proteins of interest as previously described. Anti-desmin monoclonal DE-U-10 was used at 1:1000 dilution, and secondary antibodies at 1:200 dilution [56]. 

Immunofluorescence and confocal microscopy. Immunofluorescence was performed essentially as described [56,57]. Cells grown on coverslips were fixed with 4% (*w*/*v*) paraformaldehyde, permeabilized with 0.1% (*v*/*v*) triton X-100, and blocked with 1% (*w*/*v*) bovine serum albumin in PBS (blocking solution). Antibodies were diluted in a blocking solution. Polyclonal anti-desmin from Abcam and secondary antibody horse anti-rabbit-Alexa 488 conjugate from Vector Laboratories were used at 1:200 dilution. For f-actin staining, cells were incubated with 0.25 μg/mL phalloidin-tetramethylrhodamine B isothiocyanate (phalloidin-TRITC) (Merck) in blocking solution. Nuclei were counterstained with DAPI. Coverslips were mounted with Fluorsave (Calbiochem, Merck). Images were acquired on Leica SP5 or SP8 confocal microscopes. Single sections were taken every 0.5 µm, and individual sections or overall projections are shown, as indicated. Bars are 20 µm, unless indicated otherwise.

Image analysis. Transient transfection of desmin in H9c2 cells led to the distribution of the protein in extended networks in the majority of cells, although desmin condensations with an apparent diffuse content could be appreciated in cells with the highest expression levels (less than 20% of the cells). For analysis of the effects of H_2_O_2_ and 15d-PGJ_2_ on desmin network morphology, transiently transfected H9c2 cells showing extended desmin filaments under non-saturation conditions were used as a standard to define the range of acquisition parameters. The area of the cell covered by detectable filaments under these conditions was measured with Image J and normalized by the total cell area as determined by staining of f-actin. The proportion of cells showing filament condensations or normal morphology was assessed by visual inspection of multiple randomly selected fields from at least three experiments, totaling from 50 to 100 cells for assessment of the effect of 15d-PGJ_2_. For analysis of the effects of chemical hypoxia, the proportion of cells showing detectable filaments or bearing any kind of condensation or aggregates was counted by visual inspection of randomly selected fields from three independent experiments totaling at least 300 cells per experimental condition. Analysis of desmin distribution in SW13/cl.2 cells treated with H_2_O_2_ was performed by visual inspection of randomly selected fields from three independent experiments, totaling at least 270 cells per experimental condition.

Statistical analysis. All experiments were repeated at least three times. For comparisons between two conditions of interest, the Student’s *t*-test for unpaired samples was used, and results were considered significant when *p* < 0.05.

## 3. Results

### 3.1. Cys333 of Desmin Is a Selective Target for Oxidants and Electrophiles In Vitro

**Figure 2:**
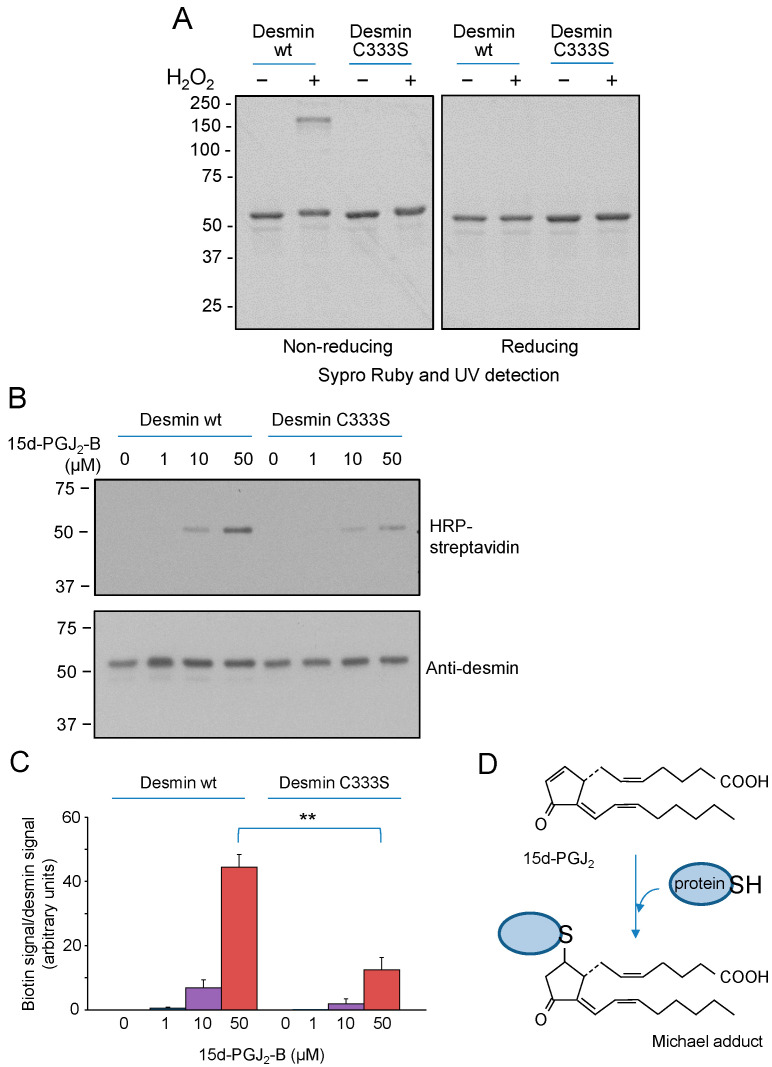
Modification of desmin by oxidants and electrophiles in vitro. (**A**) Purified desmin (wt or C333S) at 4 µM final concentration was incubated with 1 mM H_2_O_2_ in 5 mM Pipes, pH 7.0, and 0.1 mM DTT for 1 h at room temperature. Aliquots of the incubation mixtures containing 1 µg of protein were analyzed by gel electrophoresis under non-reducing or reducing conditions, as indicated, and the mobility of desmin was assessed by staining with Sypro Ruby and UV detection. The results are representative of four assays with similar results. (**B**) Desmin wt or C333S were incubated with the indicated concentrations of 15d-PGJ_2_-B for 1 h at room temperature. Aliquots of the incubation mixtures were analyzed by SDS-PAGE and transferred to membranes. Incorporation of 15d-PGJ_2_-B was estimated from the biotin signal, detected with HRP-streptavidin. Total desmin was detected by western blot. (**C**) The graph displays the ratio of the biotin signal corrected by the desmin signal. Results are average values ± SEM from four assays. ** *p* < 0.01 by Student’s *t*-test. (**D**) The scheme depicts the structure of 15d-PGJ_2_ and that of the Michael adduct of this compound with a thiol group in a protein.

The single cysteine residues of the type III intermediate filaments vimentin and GFAP play important roles in sensing oxidative and electrophilic stress [8,43,44]. To explore whether the equivalent cysteine residue in desmin, C333, plays a similar role, we first studied its modification by several compounds in vitro. Treatment of purified desmin wt with H_2_O_2_ resulted in the formation of a main oligomer of apparent molecular weight > 150 kDa, as revealed by electrophoresis under non-reducing conditions (Figure 2A). The proportion of the oligomer varied between assays from 20 to 40% of the amount of monomer. This oligomer was not detectable under reducing conditions, that is, in the presence of the disulfide breaking agent ꞵ-mercaptoethanol or in samples from H_2_O_2_-treated desmin C333S (Figure 2A). Notably, this oligomer is compatible with a disulfide-bonded dimer of desmin, given the fact that dimers of type III intermediate filament proteins, namely the highly homologous proteins vimentin and GFAP, display an apparent size much higher than that expected from the mass of the protein (monomer apparent size ~54 kDa, dimer apparent size ~140–200 kDa) [42,53,58]. This has been previously attributed to the high proportion of coiled coil structure in these proteins and the extended conformation of the cysteine-bonded dimer [58].

Next, we explored the modification of desmin by the electrophilic lipid mediators known as cyclopentenone prostaglandins (cyPG) by employing a biotinylated analog of 15-deoxy-Δ^12,14^-PGJ_2_ (15d-PGJ_2_-B). These electrophilic eicosanoids are known to form Michael adducts with thiol groups in proteins [59], including vimentin and GFAP [41,42]. We observed the incorporation of the biotin signal into the monomeric protein, which was resistant under reducing conditions, indicating that 15d-PGJ_2_-B formed an adduct with desmin. Adduct formation occurred preferentially by interaction with C333 (Figure 2B), as indicated by the significantly higher incorporation of the biotinylated cyPG into the wt protein in comparison with the desmin C333S mutant (approximately 3.5-fold, Figure 2C). No alterations in desmin electrophoretic mobility were detected under these conditions. The structure of 15d-PGJ_2_ and its Michael adduct with a cysteine residue in a protein are shown in Figure 2D.

**Figure 3:**
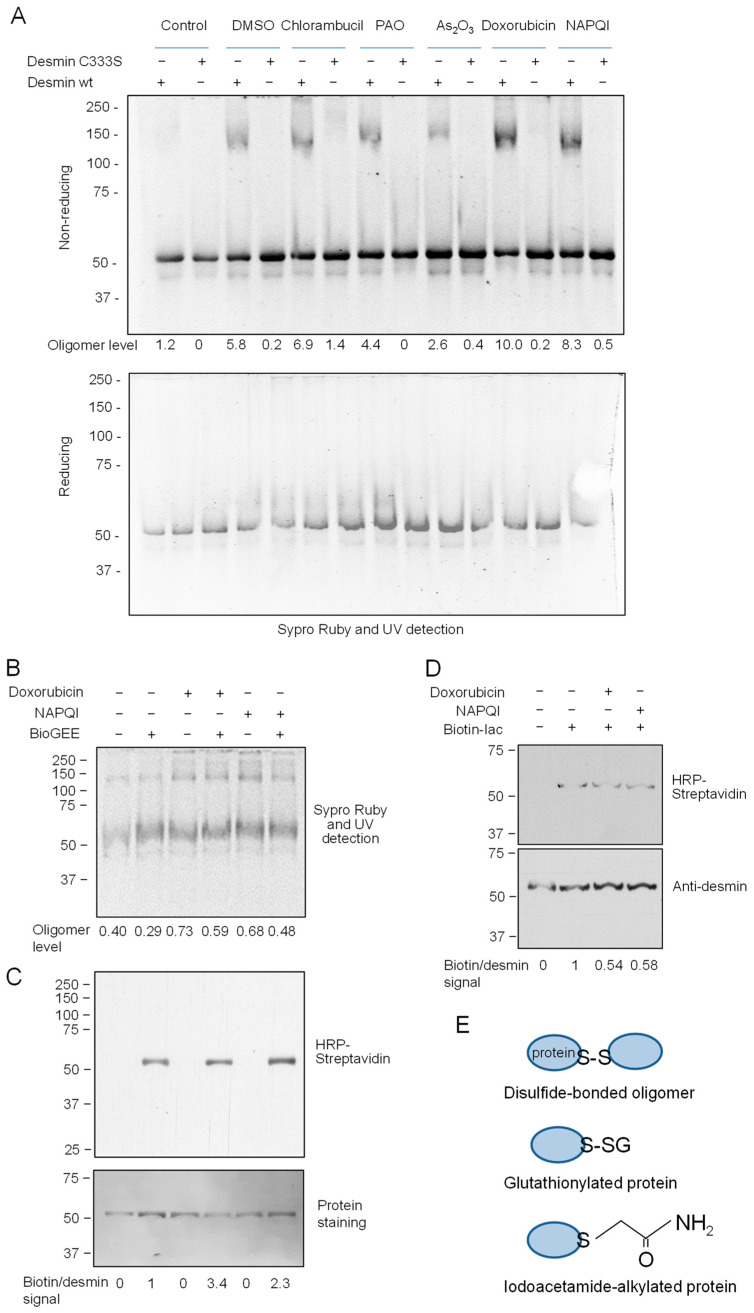
Effect of several drugs on desmin modifications in vitro. (**A**) Desmin wt or C333S was incubated in the presence of chlorambucil, phenylarsine oxide (PAO), As_2_O_3_, doxorubicin, or *N*-acetyl-*p*-benzoquinone imine (NAPQI), as indicated. Incubations were carried out for 2 h at 37 °C in the presence of a 50 µM final concentration of each compound dissolved in DMSO, except for As_2_O_3_, which was used at 500 µM and was dissolved in water. Aliquots from the incubations were analyzed by SDS-PAGE under non-reducing (**upper** gel) and reducing conditions (**lower** gel), and the mobility of the protein was assessed as in Figure 1. The results are representative of at least three assays with similar results. (**B**,**C**) Desmin wt was incubated with doxorubicin or NAPQI, as in (**A**), in the presence or absence of a 1 mM final concentration of biotinylated glutathione (BioGEE). Aliquots of the incubation were analyzed by SDS-PAGE under non-reducing conditions, followed by gel staining with Sypro Ruby for detection of total protein (**B**) or protein transfer to membranes (**C**) for detection of biotin incorporation with HRP-conjugated streptavidin (**upper** image) and total protein by GelCode staining of the blot (**lower** image). (**D**) Desmin was preincubated with doxorubicin or NAPQI as in (**A**), after which 20 µM biotinylated iodoacetamide (Biotin-Iac) was added, and incubation was continued for 30 min at room temperature in the dark before analysis of biotin incorporation as in Figure 2B. In (**A**,**B**) oligomer level, indicates the ratio between desmin oligomeric and monomeric bands of the images shown in arbitrary units. In (**C**,**D**), the ratio of the biotin signal corrected by the desmin signal in the experimental conditions illustrated is given under the blots. (**E**) Scheme depicting the modifications detected upon the various treatments, namely, disulfide bonded dimers in the presence of various drugs, glutathionylated desmin in the presence of BioGEE both in the absence and presence of drugs, and iodoacetamide-alkylated desmin, which is decreased by preincubation with drugs.

Interestingly, besides endogenous reactive species, numerous drugs and/or their metabolites can promote oxidative conditions both in vitro and in cells and/or form mono- or bis-adducts with proteins, in particular cysteine residues, a process that can be involved in their mechanism of action or in the development of adverse effects [60,61,62,63]. Therefore, we incubated desmin with several drugs, including the tyrosine kinase inhibitor phenylarsine oxide, the paracetamol metabolite NAPQI, and the antitumoral drugs As_2_O_3_, doxorubicin, and chlorambucil, and monitored its electrophoretic mobility under non-reducing and reducing conditions (Figure 3A).

Analysis under non-reducing conditions showed that most of the compounds used increased the formation of a ~150 kDa desmin oligomer with respect to that observed after incubation with the corresponding vehicle (DMSO for all compounds except for As_2_O_3_, which was dissolved in water) (Figure 3A, upper gel). In particular, As_2_O_3_, doxorubicin, and NAPQI elicited clear increases in oligomerization with respect to incubation with their respective vehicles. In all cases, oligomer formation required the presence of desmin single cysteine residue, since it was considerably decreased and virtually undetectable when desmin C333S was employed. Moreover, the oligomers formed were basically reversed under reducing conditions, suggesting the involvement of disulfide bonds in their formation (Figure 3A, lower gel). The formation of disulfide bonds in the presence of drugs could be due to a depletion of free thiols in desmin and/or in the antioxidants present in the incubation (i.e., DTT). In cells, the most important thiol-based antioxidant molecule is glutathione. Interestingly, incubation of desmin in the presence of an excess of glutathione (in the form of the biotinylated analog BioGEE) moderately decreased the formation of desmin oligomeric species, as evidenced by total protein staining (Figure 3B). Moreover, this assay allowed for the detection of desmin glutathionylation, which readily occurred upon incubation with BioGEE in vitro under control conditions (Figure 3C) and moderately increased in the presence of doxorubicin or NAPQI, as evidenced by the incorporation of biotin into the desmin monomer. As stated above, in addition to eliciting oxidative conditions, certain drugs, including NAPQI, can directly form adducts with cysteine residues [60,62,63]. Although here we have not directly assessed the formation of desmin-drug addicts or of other oxidative modifications at cysteine, such as the formation of sulfenic, sulfinic, or sulfonic groups, all these modifications would reduce the availability of free thiol residues in desmin. Indeed, we confirmed that pretreatment with doxorubicin or NAPQI diminished the incorporation of the cysteine reagent biotinylated iodoacetamide into desmin in vitro (Figure 3D), corroborating a decreased availability of free thiol groups under these conditions.

Taken together, these results indicate that the cysteine residue of desmin is an important target for in vitro modification by oxidants, electrophilic mediators, and drugs. Moreover, in accordance with previous observations [45,49], our results indicate that C333 can be the target of various modifications, some of which are schematized in Figure 3E, and that can include adduct and mixed disulfide formation.

### 3.2. H_2_O_2_ Disrupts Cellular Desmin Organization and This Is Attenuated in a C333S Desmin Mutant

**Figure 4:**
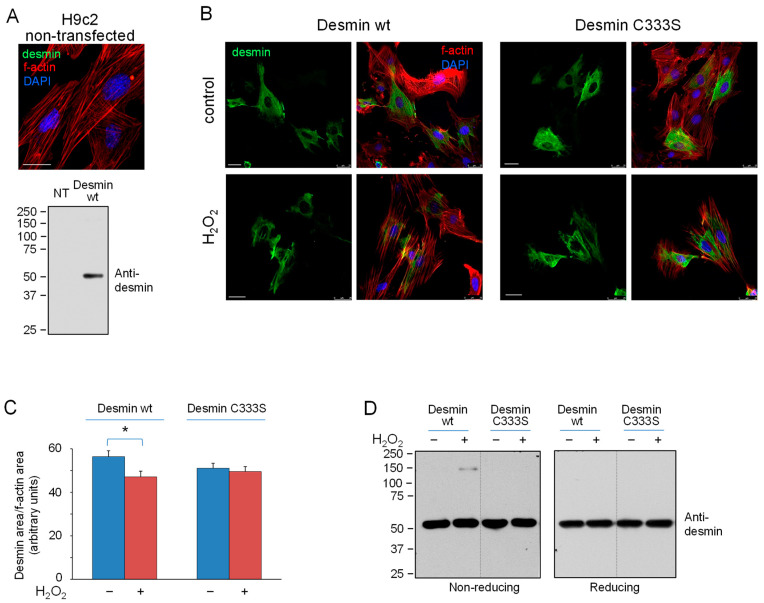
Effect of H_2_O_2_ on desmin wt or C333S network organization and oligomerization in H9c2 cardiomyoblasts. Cells were transfected with desmin wt or C333S and treated with 1 mM H_2_O_2_ for 2 h, as indicated. (**A**) Non-transfected H9c2 cells show undetectable levels of desmin either by immunofluorescence (**upper** panel) or by western blot (**lower** panel). NT, non-transfected cells; Desmin WT, cells transfected with desmin wt. (**B**) Cells grown on coverslips were fixed and stained for desmin and f-actin, and nuclei were counterstained with DAPI. Overall projections showing the distribution of desmin (**left** images) and the overlays with f-actin and DAPI (**right** images) are shown. Bars, 25 µm. (**C**) The graph displays the ratios between the area of the cells with detectable desmin filaments and that occupied by f-actin. The results shown are average values from at least 15 determinations ± SEM. * *p* < 0.05 by Student’s *t*-test. (**D**) Cells on cell culture wells were lysed, proteins were analyzed by SDS-PAGE under non-reducing or reducing conditions, as indicated, and desmin was detected by western blot. The results are representative of four experiments. Dotted lines indicate where lanes from the same gel have been cropped.

In view of the above results, we assessed whether oxidants modulate desmin organization in cells in a manner dependent on the presence of the single cysteine residue. For this, H9c2 cardiomyoblasts, which do not express detectable levels of desmin (Figure 4A), were transiently transfected with desmin wt or C333S, and the organization of the desmin network was monitored by immunofluorescence. In control cells, desmin wt formed regular, long filaments, covering a significant area of the cell, as estimated from f-actin staining (Figure 4B). Treatment with a bolus concentration of H_2_O_2_ elicited a characteristic disruption of desmin organization, with the appearance of filaments more entangled and more concentrated in the perinuclear area, frequently forming condensed bundles. Thus, the area of the cell displaying desmin wt filaments detectable under non-saturating conditions was significantly decreased after H_2_O_2_ treatment (quantified in Figure 4C). Interestingly, this morphological alteration was attenuated in cells transfected with desmin C333S, in which H_2_O_2_ did not elicit a significant retraction of detectable filaments. Calculation of the relative changes in desmin area confirmed a 17% retraction of desmin wt filaments vs. a 3% retraction of desmin C333S filaments in response to treatment with H_2_O_2_ (Figure 4C). Of note, desmin C333S filaments tended to cover a smaller cellular area than desmin wt under control conditions. Remarkably, monitoring of f-actin indicated that H_2_O_2_ also elicited a reorganization of actin into more defined stress fibers, along which desmin bundles could be frequently detected (Figure 4B).

Analysis of desmin by SDS-PAGE under non-reducing conditions revealed the formation of oligomeric species of apparent size > 150 kDa, analogous to those observed in vitro in cells treated with H_2_O_2_. These oligomers were reversed under reducing conditions and were not detectable in cells transfected with desmin C333S, indicating that they correspond to disulfide-bonded desmin dimers (Figure 4D). Remarkably, no alterations of the desmin monomer or signs of degradation were detected under these conditions.

### 3.3. 15d-PGJ_2_ Elicits Desmin Network Alterations, Which Are More Intense in the wt Protein

**Figure 5:**
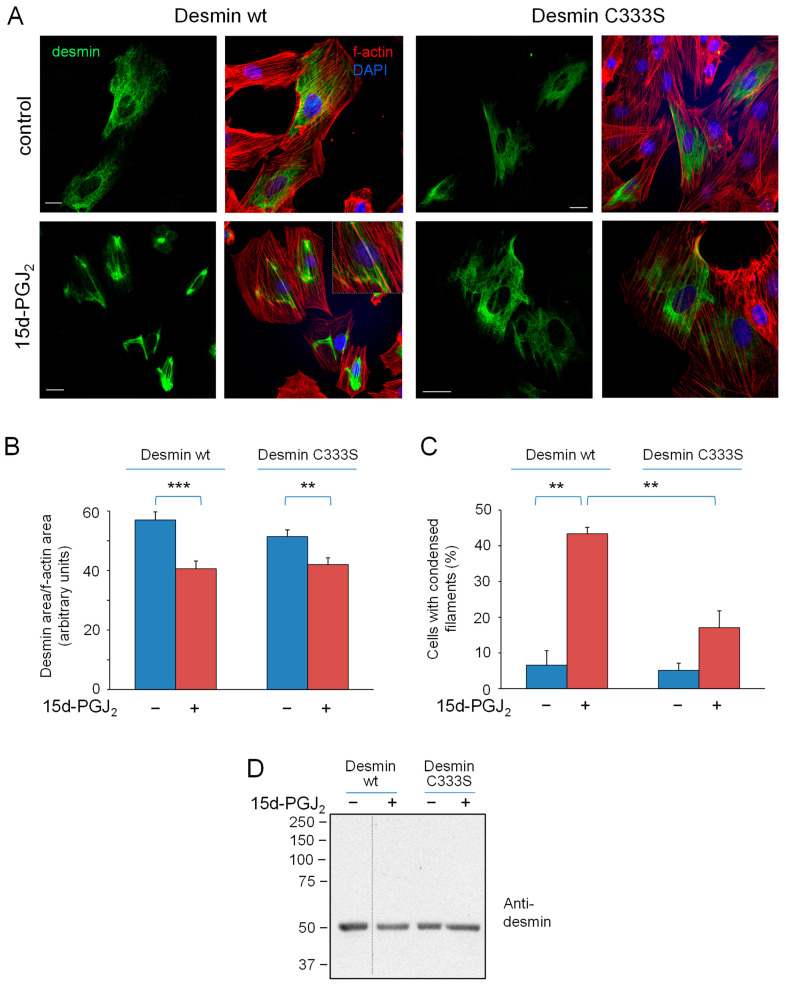
Effect of 15d-PGJ_2_ on desmin network organization and protein levels in H9c2 cardiomyoblasts. Cells transfected as above were treated with 10 µM 15d-PGJ_2_ for 2 h. (**A**) Distribution of desmin and f-actin was assessed as in Figure 3. (**B**) Ratios between the cell areas covered by detectable structures of desmin and actin. Results shown are average values from at least 18 determinations ± SEM. *** *p* < 0.001, ** *p* < 0.01 by Student’s *t*-test. (**C**) Proportion of cells showing desmin accumulations. Results are average values ± SEM from four different assays totaling between 50 and 100 cells per experimental condition. (**D**) Lysates from cells treated as in (**A**) were analyzed by gel electrophoresis under non-reducing conditions, and desmin was detected by western blot. Results are representative of four experiments. The dotted line indicates where lanes from the same gel have been cropped.

Next, we explored the effect of the electrophilic mediator 15d-PGJ_2_ on desmin organization in cells. 15d-PGJ_2_ provoked a marked rearrangement of the desmin network, consisting of a condensation of filaments, which frequently appeared near the nucleus or in dense bundles and sometimes aligned over actin stress fibers and/or became more entangled with them (Figure 5A). 15d-PGJ2-elicited retraction of desmin wt from the cell periphery appeared to be more intense than that induced by H_2_O_2_ treatment. Although desmin C333S filaments also suffered a significant retraction in response to treatment with 15d-PGJ_2_, it was less marked than that of the wt (28% vs. 18% average retraction in wt and C33SS, respectively, quantitated in Figure 5B). Moreover, the proportion of cells displaying filament bundles of juxtanuclear condensations was significantly lower in cells expressing desmin C333S than in those bearing desmin wt (42% and 17% of cells showed filament bundles of condensations in cells expressing desmin wt and C333S, respectively, Figure 5C). Analysis of cell lysates from cells treated with 15d-PGJ_2_ under non-reducing conditions did not reveal any noticeable alterations in desmin electrophoretic mobility or levels (Figure 5D), implying that no detectable disulfide-bonded desmin dimers were generated by 15d-PGJ_2_ treatment.

Taken together, these results show that expression of desmin C333S in H9C2 cells affords partial protection of the cytoplasmic intermediate filament network from oxidants and electrophiles. It should be considered that these cells express vimentin, the single cysteine of which is present in all conditions and could be targeted and perturbed by the agents used, which could attenuate the protective effect of cysteine-deficient desmin on network disruption, as vimentin is known to form shared filament networks with desmin in certain tissues and cell types [44].

### 3.4. Chemical Hypoxia Elicits Desmin Rearrangement Dependent on C333

**Figure 6:**
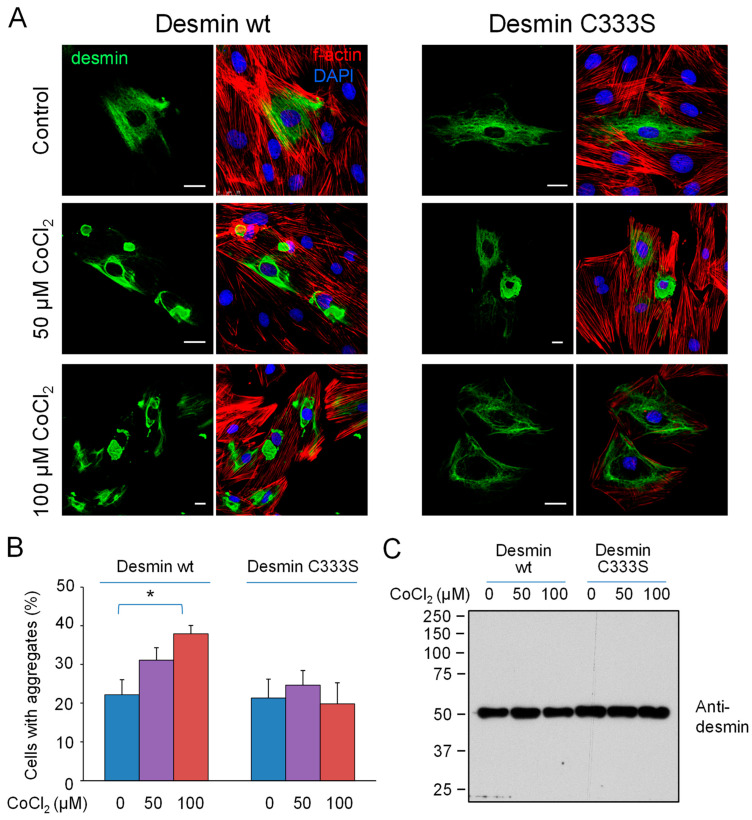
Effect of chemical hypoxia on the organization of desmin wt or C333S in H9c2 cells. Cells were transfected with desmin wt or C333S, as indicated, and treated with the specified concentrations of CoCl_2_ for 24 h. (**A**) Cells were fixed and processed for detection of desmin (immunofluorescence), f-actin (phalloidin-TRITC staining), and nuclei (DAPI). (**B**) The proportion of cells showing any type of desmin condensation or aggregate was obtained by visual inspection from three different experiments. Results shown are average values ± SEM. * *p* < 0.05. Bars, 20 µm. (**C**) Lysates from cells treated as in (**A**) were analyzed by SDS-PAGE under non-reducing conditions and immunoblotted with an anti-desmin antibody. Results are representative of three assays with similar observations.

A pathophysiological situation critically associated with oxidative stress is hypoxia. Production of reactive oxygen species and subsequent oxidative stress are adaptation mechanisms to both acute and chronic hypoxia [64]. However, in organs with high demand for oxygen, such as the heart, these events can lead to cardiomyocyte loss, cardiac remodeling and heart failure [35,65]. To explore the potential impact of hypoxia-like conditions on desmin organization, we employed a broadly used approach to elicit chemical hypoxia by treating cells with CoCl_2_ [66] (Figure 6). This model shares numerous features of low oxygen hypoxia, including the induction of HIF-1α and the formation of ROS [67], as well as various transcriptional changes [68,69,70,71] in diverse experimental systems, although there are also differential features (see [66] for review). Treatment of H9c2 cardiomyocytes expressing desmin wt with CoCl_2_ elicited a marked reorganization of desmin wt consisting in a retraction or disruption of the extended filament network, coinciding with an increase in the appearance of various forms of filament condensations and aggregates (Figure 6A) in a significant proportion of cells (quantitated in Figure 6B). Remarkably, the effects of CoCl_2_ treatment were markedly attenuated in cells transfected with the cysteine-deficient desmin mutant, C333S (Figure 6A). Indeed, CoCl_2_ did not appear to increase aggregate formation of the mutant protein, and extended desmin filaments were preserved in a high proportion of cells expressing desmin C333S (Figure 6B). Notably, under these conditions, no alterations in desmin electrophoretic mobility were observed by non-reducing SDS-PAGE, indicating that neither disulfide formation nor appreciable degradation were occurring to a significant extent (Figure 6C).

### 3.5. Importance of C333 for Desmin Organization in Cells under Non-Stress Conditions

**Figure 7:**
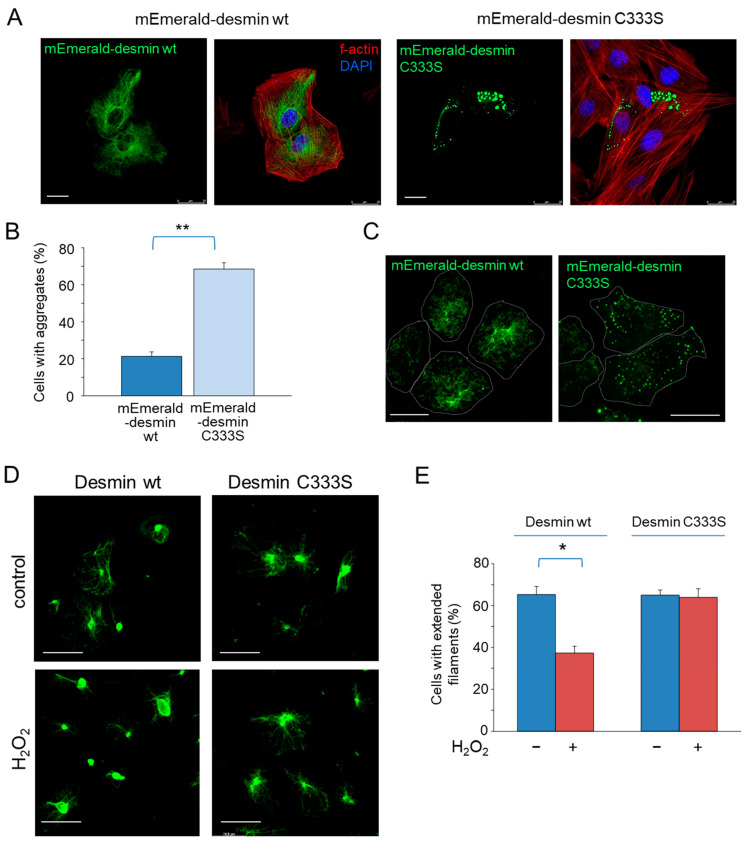
Importance of C333 in desmin assembly in several cellular models. (**A**) H9c2 cardiomyoblasts were transfected with mEmerald-desmin wt or C333S, as indicated. The morphology of the desmin network was assessed 48 h later by direct fluorescence visualization; f-actin was stained with Phalloidin-TRITC, and nuclei were counterstained with DAPI. The proportion of cells displaying desmin aggregates is shown in (**B**). Results are average values ± SEM from three different experiments. ** *p* < 0.01 by Student’s *t*-test. (**C**) SW13/cl.2 cells were transfected with the indicated plasmids, and the distribution of the desmin fluorescent construct was assessed by confocal microscopy. Images are representative of four assays with similar results. (**D**) SW13/cl.2 cells expressing desmin wt or C333S were treated with H_2_O_2_, as indicated. The morphology of the desmin network was assessed by immunofluorescence. The proportion of cells showing extended desmin filaments is shown in (**E**). Results are average values ± SEM from three independent experiments. * *p* < 0.05 by Student’s *t*-test.

Taken together, the results shown above suggest that desmin C333S is more resistant than the wt protein to a variety of oxidative insults. Nevertheless, under our conditions, desmin C333S tends to form less extended networks in H9c2 cardiomyoblasts, suggesting that C333 is important for desmin organization. To substantiate this possibility, we employed several strategies. We have previously observed that expression of vimentin or GFAP as fusion constructs with fluorescent proteins, such as GFP or mCherry, at their N-terminus magnifies the assembly defects due to mutations of their single cysteine residues, C328 and C298, respectively [41,42]. Therefore, we first compared the distribution of mEmerald-desmin wt and C333S in H9c2 cells upon transient transfection. mEmerald-desmin wt led to the formation of long filaments in the majority of cells, whereas a small proportion of the cell population showed mEmerald-desmin dots or small aggregates (Figure 7A).

In sharp contrast, expression of mEmerald-desmin C333S resulted in the abnormal assembly of this construct, with the appearance of desmin dots in a high proportion of cells (quantified in Figure 7B). Our previous work indicates that defects in network formation in other type III intermediate filament mutants are better evidenced when expressed in cells deficient in cytoplasmic intermediate filaments. Therefore, we next assessed the organization of desmin wt and C333S constructs in the adrenal carcinoma cell line SW13/cl.2, which lacks a cytoplasmic intermediate filament network [54]. In these cells, mEmerald-desmin wt formed regular squiggles and short filaments, whereas mEmerald-desmin C333S led mostly to multiple bright dots or accumulations of heterogeneous size and distribution, which in some cells coexisted with faint squiggles appreciable in the cytoplasm. (Figure 7C). Therefore, the presence of C333 appears to be important for optimal mEmerald-desmin organization in this cellular model. In turn, untagged desmin wt or C333S led to the formation of mixed assemblies consisting of extended filaments and accumulations near the center of the cell (Figure 7D, upper images). Next, we took advantage of this simple model system to confirm the role of C333 modification in desmin remodeling in response to oxidative stress without the contribution of other cytoplasmic intermediate filaments. Treatment with 1 mM H_2_O_2_ for 1 h led to an increase in the proportion of cells showing intense accumulations of desmin at the center of the cell and a concomitant decrease in the proportion of cells bearing extended filaments. This effect was significantly attenuated in cells expressing desmin C333S (Figure 7D, lower images, quantitated in Figure 7E).

Taken together, these results indicate that desmin C333S shows some assembly or organization defects with respect to desmin wt in cells. Nevertheless, this mutant appears to be less susceptible to alteration by oxidative stress than desmin wt.

## 4. Discussion

Type III intermediate filament networks are not merely structural elements but act as integrators of cytoskeletal responses as well as sensors of the cellular redox context and targets of reactive species. Desmin, the type III intermediate filament expressed in muscle cells, is emerging as a key player in muscle pathophysiology. Desmin assembly and function are subjected to tight regulation through various posttranslational modifications, such as phosphorylation, ubiquitylation, ADP ribosylation, glycation, and nitration [72,73]. These modifications mainly target serine, threonine, tyrosine and arginine residues [72,73]. In turn, the present work focuses on the single cysteine residue of desmin, C333. The results herein described show that reactive species, as well as conditions associated with oxidative stress, can elicit desmin oxidative modifications or form adducts, for which desmin C333 appears to be an important target. Moreover, these agents induce morphological alterations of the desmin network in cells, which depend in part on the presence of C333. Lastly, the presence of this residue appears to be important for desmin organization under basal conditions, which could involve the free thiol form or proteoforms compatible with assembly.

We have observed that several agents, including oxidants, electrophiles, and drugs or drug metabolites, elicit modifications of desmin in vitro, some of which are readily detectable as disulfide-bonded desmin oligomers by gel electrophoresis under non-reducing conditions, such as that elicited by H_2_O_2_ treatment. The adduct formed with 15d-PGJ_2_ is resistant under reducing conditions, can be easily detected by use of the biotinylated analog, and displays selectivity for the cysteine residue, although at high concentrations, cyclopentenone prostaglandins may target other residues [43,74]. Of the other compounds tested, PAO, As_2_O_3_, and chlorambucil have been reported to be able to react simultaneously with two cysteine residues [60,75], which could be involved in the appearance of the desmin oligomer. In contrast, other cysteine-reactive drugs including NAPQI are known to preferentially form single adducts with thiols [76,77], for which the appearance of the reversible desmin oligomer could be due to indirect mechanisms. In turn, our results indicate that the cardiotoxic antineoplastic drug doxorubicin can also promote desmin oligomerization under non-reducing conditions. Precisely, NAPQI and doxorubicin are the drugs that appear to more potently induce desmin reversible oligomerization in vitro. Notably, besides disulfide-bonded desmin dimers, in vitro glutathionylation of desmin can also occur in the presence of glutathione, as evidenced by the use of a biotinylated glutathione analog (BioGEE). This indicates that multiple modifications can occur on desmin C333 and potentially compete, depending on the redox conditions and the availability of antioxidants. Consistent with the occurrence of modifications at cysteine, a decrease in iodoacetamide incorporation and, therefore, in the content of free thiol availability occurs upon desmin incubation with drugs. Additional cysteine modifications, not assessed in this work, could potentially contribute to this effect, including other oxidized states of the cysteine residue, such as oxidation to sulfenic, sulfinic, or sulfonic acids, or the formation of drug adducts at cysteine, for instance in the case of NAPQI.

Interestingly, treatment of cells with agents inducing oxidative stress elicits alterations of the desmin network, often appearing as a retraction of filaments towards the nucleus and showing variable degrees of disorganization or condensation into aggregates. The juxtanuclear condensation of desmin filaments bears similitude with that observed for other type III intermediate filaments, namely, vimentin and GFAP, exposed to electrophilic agents [41,42]. In this context, it is interesting to note that remodeling of both vimentin and GFAP in response to electrophiles is highly dependent on the presence of their single cysteine residues [10,41,42]. Importantly, these residues are evolutionary conserved, and accumulating evidence suggests that they are redox sensitive and play a role in network organization and in cytoskeletal crosstalk. Here we have observed that treatment of H9c2 cells with H_2_O_2_ elicits the formation of a disulfide bonded dimer. This oligomer was not detected after treatment with 15d-PGJ_2_ or CoCl_2_. This could be due to the occurrence of other cysteine modifications, such as the formation of adducts in the case of 15d-PGJ_2_, and/or of irreversible oxidative modifications, which would preclude disulfide bond formation. As expected, desmin disulfide oligomerization does not occur in cells expressing the desmin C333S mutant. Moreover, desmin alterations elicited by various oxidants are partially but significantly attenuated in H9c2 cells transfected with desmin C333S. Indeed, as H9c2 cardiomyoblasts also express vimentin [78,79], a partial protective effect of mutant desmin could be foreseen since endogenous vimentin would still be susceptible to the effect of electrophiles, and could influence the impact of these compounds on the intermediate filament network. Nevertheless, the lower susceptibility of desmin C333S to oxidative stress-elicited network remodeling has also been evidenced in SW13/cl.2 cells, which do not express other cytoplasmic intermediate filaments, thus highlighting the role of this cysteine residue in network reorganization. Therefore, although several chaperones or heat shock proteins, including αB-crystallin and Hsp27, act as regulators of desmin folding, polymerization, and correct filament localization [13,80,81], direct modification of desmin appears to play an important role in this cell model.

Moreover, analogously to GFAP and vimentin, C333 in desmin appears to be important for the normal assembly of the filaments or the network. On the one hand, filaments formed by desmin C333S appear to extend less towards the cell periphery than the wt filaments when transfected in cells. Furthermore, expression of desmin C333S as a fusion protein with a fluorescent tag at its N-terminus further hampers its assembly in SW13/cl.2 cells, thus amplifying the assembly defect, as previously reported for the corresponding vimentin and GFAP mutants [41,42]. Interestingly, certain cysteine modifications, such as S-sulfhydration, can occur under physiological conditions, and together with other reversible modifications, such as sulfenylation or disulfide formation, they are considered regulatory and/or protective mechanisms against more deleterious oxidations [36,82,83]. Nevertheless, to the best of our knowledge, it is not currently clear whether desmin can be sulfhydrated under controlled conditions or if certain cysteine modifications are compatible with desmin function or assembly.

An important question may be the function or consequences of departmental reorganization or disruption. It has been proposed that desmin misfolding in response to chemical or mechanical stress, such as that caused by increased workload, could represent a defense mechanism, helping to dissipate stress and protecting organelles (i.e., sarcomeres and mitochondria) important for cardiomyocyte function [19,27]. In this context, attenuating desmin disruption by mutating or protecting the cysteine residue could exert a deleterious effect. Indeed, a recent report indicates that the outcome of ischemia/reperfusion in mice is worse in mice transduced with cysteine-deficient forms of desmin, C328S or C328A [46]. On the other hand, excessive desmin aggregation could exert deleterious consequences on the muscle cells, and in this situation, preventing excessive desmin aggregation could be beneficial. In this context, it should be noted that a propensity for desmin to form aggregates has been reported [84,85]. This is enhanced by certain modifications and mediated by particular regions of the protein that can adopt amyloid folds [86]. Since desmin aggregates have been shown to induce further desmin misfolding in vitro [85], a similar phenomenon in cells could contribute to pathogenic mechanisms in cardiac and skeletal muscle diseases.

Considering several lines of evidence, it could be hypothesized that oxidative stress arising from several muscle diseases, including desminopathies, could contribute to desmin C333 oxidation, which could initially mediate desmin protective reorganization, but when excessive, it could contribute together with other posttranslational modifications, such as phosphorylation and cleavage, to uncontrolled aggregation and deleterious effects. Indeed, several situations associated with oxidative stress result in desmin aggregation [31,46], including the effects of CoCl_2_ shown herein, whereas certain antioxidants have shown protective effects. In particular, N-acetyl-cysteine has been reported to prevent oxidative stress-induced desmin aggregation in cellular models of desminopathy [31], and tocopherols decreased the proportion of C2C12 myoblasts displaying spontaneous aggregates upon transfection of a GFP-Desmin D399Y mutant [32]. Nevertheless, antioxidants could also exert general beneficial effects, for instance, by protecting mitochondria from excessive damage. Considering that desmin degradation appears to be the trigger for muscle wasting, strategies aimed at preventing desmin degradation are being explored as therapeutic approaches for desminopathies and muscle atrophy. It should be noted that, as the aim of our study was to monitor acute desmin reorganization, we used short incubation times for most of our study. Under our conditions, we have not observed desmin degradation, even after treatment of cells with CoCl_2_. Although we have evidenced an attenuation of CoCl_2_-elicited desmin aggregation in cells expressing desmin C333S, further work will be needed to assess whether this implies a protective or deleterious role of C333 oxidation in the long term.

Intermediate filaments in general and type III in particular are considered stress proteins, playing protective roles against various kinds of stress [9,87]. Desmin has been shown to be critical for stress transmission and signaling in skeletal muscle [7]. Vimentin plays protective roles against hypotonic shock [88], and in response to mechanical stress [89]. The marked reorganization of filaments towards the nucleus occurring upon stress has also been proposed to play a protective role by concentrating associated chaperones and shielding organelles [90]. The conserved cysteine residues in these proteins are emerging as hot spots for modification by oxidants and electrophiles [8]. However, whether these modifications play a role in the protective effects of type III intermediate filaments under stress is still not clear. In this context, oxidative stress-induced nuclear alterations appear to be more severe in cells expressing cysteine-deficient lamins [91]. Moreover, we have observed that filamentous vimentin cysteine-deficient mutants blunt the induction of actin stress fibers by certain electrophiles, thus precluding the full response of cells to these kinds of stress [10]. Similarly, a C328S vimentin mutant is less competent than the wild type at forming aggresomes upon inhibition of the proteasome [41], which could imply a reduced function in the elimination of misfolded proteins. Tools and approaches recently developed should help clarify the importance of cysteine modifications in the management of stress by type III intermediate filament proteins.

## 5. Concluding Remarks and Perspectives

In summary, the results presented herein indicate that, analogously to vimentin and GFAP, the single cysteine residue of desmin plays an important role in the formation of filaments and/or in the organization of the desmin network. As with these proteins, the assembly defects of desmin C333S are more evident in cells devoid of an endogenous cytoplasmic intermediate filament network and are amplified in fluorescent fusion constructs. Moreover, C333 appears to be important for the remodeling of desmin in response to situations associated with oxidative stress in cellular models, since desmin reorganization is attenuated in cells expressing the C333S mutant. Therefore, this work calls attention to the importance of this residue in desmin regulation. Our results also suggest a relationship between the presence of C333 and the susceptibility of desmin to bundling and/or aggregation. Given the frequent association of desminopathies with oxidative stress, the potential contribution of cysteine modifications to desmin aggregation or dysfunction needs to be addressed. These studies set the basis for assessing the impact of specific cysteine modifications, i.e., the behavior of defined desmin proteoforms in filament assembly in vitro. This could help ascertain whether there are modifications compatible with function, which hypothetically could play a protective role in cells. Nevertheless, exploring the presence and cellular consequences of specific desmin modifications in the cellular context would be more challenging. Given the fact that oxidative stress targets numerous proteins, defining a role for desmin would probably require the use of oxidation-resistant constructs such as those employed here in various experimental models deficient in desmin and/or vimentin. On the other hand, cysteine oxidation mimics could also be employed. Therefore, structure-function studies could be performed in vitro and in cells. This could help elucidate whether desmin cysteine modifications have a regulatory potential that could be used to modify desmin-associated pathological conditions.

## Data Availability

The data presented in this study are currently available on request from the corresponding author.
